# The Role of Access Type and Age Group in the Breadth of Use of Patient Portals: Observational Study

**DOI:** 10.2196/41972

**Published:** 2022-12-27

**Authors:** Theophile Ndabu, Lavlin Agrawal, Raj Sharman

**Affiliations:** 1 Belk College of Business University of North Carolina at Charlotte Charlotte, NC United States; 2 State University of New York University at Buffalo Buffalo, NY United States

**Keywords:** patient portal, mobile health apps, electronic medical records, personal health record functionalities, patient satisfaction, health information, Health Information National Trends Survey, healthcare delivery, app use, electronic medical record

## Abstract

**Background:**

Health care delivery and patient satisfaction are improved when patients engage with their medical information through patient portals. Despite their wide availability and multiple functionalities, patient portals and their functionalities are still underused.

**Objective:**

We seek to understand factors that lead to patient engagement through multiple portal functionalities. We provide recommendations that could lead to higher patients’ usage of their portals.

**Methods:**

Using data from the Health Information National Trends Survey 5, Cycle 3 (N=2093), we performed descriptive statistics and used a chi-square test to analyze the association between the demographic variables and the use of mobile health apps for accessing medical records. We further fitted a generalized linear model to examine the association between access type and the use of portal functionalities. We further examined the moderation effects of age groups on the impact of access type on portal usage.

**Results:**

Our results show that accessing personal health records using a mobile health app is positively associated with greater patient usage of access capabilities (β=.52; *P*<.001), patient-provider interaction capabilities (β=.24, *P*=.006), and patient–personal health information interaction capabilities (β=.23, *P*=.009). Patients are more likely to interact with their records and their providers when accessing their electronic medical records using a mobile health app. The impacts of mobile health app usage fade with age for tasks consisting of viewing, downloading, and transmitting medical results to a third party (β=–.43, *P*=.005), but not for those involving patient-provider interaction (β=.05, *P*=.76) or patient–personal health information interaction (β=–.15, *P*=.19).

**Conclusions:**

These findings provide insights on how to increase engagement with diverse portal functionalities for different age groups and thus improve health care delivery and patient satisfaction.

## Introduction

### Background

Increased collaboration between patients and providers in the delivery of health care results in patient engagement and ultimately patient satisfaction [1-4]. Thus, hospitals and medical practices proactively involve patients in decision-making and all other aspects of care by offering them access to their personal health information (PHI) stored in electronic health records—a health care provider–facing digital copy of patients' medical records. Patients can access, share, and interact with both their medical records and their providers through PHI management tools, often called patient portals (hereinafter referred to as “portals”) [5-10]. In the United States, portals are used to meet calls from the Office of the National Coordinator (ONC) to provide patients with the capabilities to view, download, and transmit (VDT) their records to a third party of their choice [11,12]. Patients’ usage of VDT and other functionalities (exchanging messages with providers, scheduling medical appointments, etc) helps health care practitioners receive financial incentives. However, health care practitioners must meet qualifying usage thresholds set forth by the Promoting Interoperability Programs of the US Centers for Medicare and Medicaid Services [3,7,11]. Engagement with functionalities of portals improves the quality of care delivery and patient satisfaction [13-25]. Thus, understanding factors that lead to patient engagement through the use of multiple portal functionalities is warranted.

Portal usage remains low despite its benefits [7,9]. Several studies have also shown that portal usage varies on the basis of patients’ chronic disease and demographic statuses, such that patients from ethnic minorities are less likely to access and interact with their medical records electronically [6,26-31]. Previous research also shows that health information technology characteristics, such as usability and convenience, influence the use of portals [32-36]. A recent survey on consumers’ use of mobile apps and mobile browsers showed that people spend nearly 3 hours daily on mobile apps but less than an hour using mobile browsers [37]. This trend aligns with prior research that patients, including older patients, are interested in using mobile apps [31-33]. The extent to which the convenience of mobile health apps translates into the diversity of use of portal functionalities has not been studied. Building on these studies and using a nationally representative sample, we examine the role of portal access type and mobile health app use on the likelihood that patients of different age groups will use multiple portal engagement functionalities. These functionalities include VDT and tasks allowing users to interact with their PHI and providers. We further examine which functionalities benefit from each portal access type.

To the best of our knowledge, prior studies have not described or examined factors that lead to the use of diverse capabilities of portals in a nonintegrated context among patients using a mobile health app. Understanding these factors could help designers and health care practitioners facilitate both the frequency and the breadth of the use of portal functionalities by patients and also develop proper access-based intervention for underused capabilities.

### Engagement Functionalities and Patient Engagement

The ONC classifies portal engagement functionalities into 2 types based on their utility. The first type consists of capabilities that allow patients to access their medical records to perform VDT tasks. The second type of engagement functionality includes capabilities that facilitate web-based interactions between both patients and their providers and patients and their PHI. For example, interaction functionalities include capabilities such as secure messaging between the patient and provider, refilling prescriptions, and amending personal records [11]. Since the use of functionalities depends in part on their utility, the study adopts the ONC classification as well as the term engagement functionalities to describe portal capabilities that allow patients to (1) access their personal health information or (2) interact with their providers and (3) interact with their data. Similarly, we refer to patients’ use of engagement functionalities as patient engagement with their care in line with extant research [3,7,11,36].

Our study examines the role of portal access type in promoting patient engagement through the use of portal engagement functionalities. We specifically hypothesize that patients accessing their electronic medical records using a mobile health app are more likely to participate in all 3 aspects of engagement: VDT, patient-provider interaction (PPI), and patient-PHI interaction (PPHI). We also propose that the effects of access type on portal usage vary by functionality and age group. Specifically, the intensity and the breadth of the use of engagement functionalities differ on the basis of whether portal users are younger than 65 years. This study controls for demographic- and health-related behavioral variables.

## Methods

### Data Source and Study Population

We used data from the Health Information National Trends Survey (HINTS) 5, Cycle 3, collected by the National Cancer Institute between January and May 2019. HINTS 5 Cycle 3 surveys noninstitutionalized civilian US adults using a 2-stage sampling design. Data were collected from 5438 respondents out of 23,430 targeted addresses (overall response rate 30.3%) [38]. The survey methods and detailed reports have been published elsewhere [38-40]. HINTS data sets have been used in the literature to study health-related behaviors, including information seeking and sharing, patient-provider trust, and HIT adoption and use in health care [34-36,41-43]. This study focuses on patients who indicated on the survey questionnaire whether they use a smartphone health app to access their electronic medical records. We filtered survey responses to exclude those who did not know whether they had used an app and missing values for this parameter. The resulting sample consisted of 2093 observations.

### Variables

#### Predictor and Control Variables

The main predictor in this study was whether users accessed their web-based medical records using a smartphone health app. Participants who responded as having used smartphones to access their information were recoded as 1 and those who had not as 0. The study also controlled for demographic and health-related variables, including self-reported gender, income, age (younger than 65 years having been recoded as 0 and 65 years or older as 1), education, race and ethnicity, general health status, and insurance status. In addition, in line with previous HIT studies, we controlled for the chronic disease status [35,36] and propensity to search for health information on the internet [6,36,41]. The *propensity for searching health information on the internet* parameter was derived from questionnaire responses on whether respondents had used a computer, smartphone, or other electronic means to search for medical information on the internet. Survey questions and variable measurements are reported in Multimedia Appendix 1.

#### Outcome Variables

The role of app use on portal usage was examined using 3 engagement functionalities: VDT, PPI, and PPHI.

VDT scores were obtained by summing up answers to 3 dichotomous survey questions regarding the use of electronic medical records. First, respondents were asked whether they used their portals to view laboratory results and download medical records. “Yes” answers were recoded as 1 and “No” answers as 0. Second, respondents were also asked whether they had used their portals to share their electronic records with another health care provider, a family member, or a health app to manage or store the data. Those who had shared their records with any of the options were recoded as 1 and those who had not as 0. Finally, the recoded responses to the 3 questions were summed to obtain a VDT score ranging from 0 to 3.

We conceptually grouped responses to questions regarding the use of interaction functionalities into 2 categories. The first category consisted of functionalities related to care and communication convenience. For example, respondents were asked whether they requested medication refills or sent a secure message to their providers. We called this parameter PPI. The variable ranged from 0 to 2 after summing the dichotomous values of the questionnaire responses. The second parameter, PPHI, consisted of answers to questions on whether users (1) requested the correction of erroneous information in their records, (2) added information to their records to share with their health care provider, or (3) used their health records in deciding on a treatment for an illness or health condition. We used the values of the 3 items to compute the PPHI factor score, ranging from 0 to 3. The acronym VDT has been used extensively in the literature to represent the outcomes of VDT functionalities and, thus, yield little or no confusion [3,7,11]. However, this is not the case with interaction functionalities. We, therefore, used themes underlying these capabilities to represent interaction capabilities in line with extant literature [36].

### Statistical Analyses

We used descriptive statistics and performed a chi-square test to analyze the association between the demographic variables and app use for accessing medical records. Using a generalized linear model, we examined the association between app use and portal engagement functionalities and the moderating effect on respondents’ age group. Our analyses incorporated replicate weights to account for the survey design methodology. R (The R Foundation) statistical software was used to conduct the analyses. A *P* value of ≤.05 was considered significant.

### Ethical Considerations

This study uses publicly available (secondary) data; hence, institutional review board review was not required.

## Results

### Outcome Variables

PPI and PPHI were further validated with exploratory factor analysis (EFA) in line with previous research [36]. The suitability of the data for an EFA was assessed with the nonsignificant Little missing value test (^2^_68_=61, *P*=.71), indicating that the data were missing completely at random (significant Bartlett test for sphericity: ^2^_21_= 1617.25, *P*<.001; Kaiser-Meyer-Olkin measure of sample adequacy: overall index=0.74). As shown in Table 1, a survey-weighted EFA with maximum likelihood suggested 2 main themes from the questionnaire responses. A scree plot and eigenvalues (>1) supported selecting 2 themes for the analysis.

**Table 1:** Exploratory factor analysis to extract common themes from the data.

Variables	Factor loadings	
	Factor 1	Factor 2
Refill prescriptions	N/A^a^	0.45
Send secure message	N/A	0.64
Request correction in personal health information	0.37	N/A
Add info to medical records	0.81	N/A
Make care decisions	0.44	N/A

^a^N/A: not applicable.

### Descriptive Analysis

Our sample consisted of 2093 observations. As shown in Table 2, of all the respondents, only 714 (37.2%) patients reported using an app to access their portals. Females accounted for the majority of respondents (n=1186, 57.3%). The association between app use and age group variables was significant (*P*<.001), indicating that the impact of this association was not uniform across age group levels. As shown in Table 2, respondents younger than 65 years were more likely to report using an app (n=553, 88.5%) than those aged 65 years or older (n=154, 11.5%). The data also show that most respondents (n=1824, 84.1%) had more than a high school degree. Most app users earned US $50,000 or more (n=467, 64.4%) and were essentially non-Hispanic White (n=399, 62.5%). Most respondents self-reported their general health status as *good* or better (n=620, 89.4%).

**Table 2:** Demographic and health-related categorical variables.

Variables			Overall, n (%)		Access without app, n (%)		Access with app, n (%)	*P* value	
Participants			2093 (100)		1379 (62.8)		714 (37.2)	N/A^a^	
**Gender**									.55
	Female	1186 (57.3)		769 (56.3)		417 (58.9)			
	Male	771 (42.7)		512 (43.7)		259 (41.1)			
**Age group (years)**									<.001
	<65	1411 (82.8)		858 (79.4)		553 (88.5)			
	≥65	651 (17.2)		497 (20.6)		154 (11.5)			
**Education**									.78
	Less than high school	36 (1.7)		25 (1.7)		11 (1.6)			
	High school graduate	195 (14.2)		132 (15.2)		63 (12.5)			
	Some college	584 (43.2)		377 (42.4)		207 (44.7)			
	College graduate or more	1240 (40.9)		823 (40.7)		417 (41.1)			
**Income (US $)**									.26
	<20,000	173 (10.5)		112 (9.6)		61 (12.0)			
	20,000 to <35,000	169 (8.2)		105 (6.2)		64 (11.6)			
	35,000 to <50,000	233 (13.5)		174 (14.3)		59 (12.1)			
	50,000 to <75,000	382 (19.3)		255 (20.5)		127 (17.3)			
	≥75,000	945 (48.5)		605 (49.4)		340 (47.1)			
**Health status**									.86
	Poor	36 (2.4)		21 (2.3)		15 (2.6)			
	Fair	214 (8.9)		143 (9.4)		71 (8.1)			
	Good	706 (34.3)		462 (35.2)		244 (32.9)			
	Very good	825 (41.0)		551 (40.0)		274 (42.8)			
	Excellent	284 (13.3)		182 (13.1)		102 (13.7)			
**Race and ethnicity**									.14
	Non-Hispanic White	1339 (69.4)		940 (73.4)		399 (62.5)			
	Non-Hispanic Black or African American	219 (8.6)		132 (8.2)		87 (9.1)			
	Hispanic	233 (12.9)		113 (9.6)		120 (18.4)			
	Non-Hispanic Asian	96 (5.4)		56 (5.3)		40 (5.5)			
	Non-Hispanic other	73 (3.8)		46 (3.4)		27 (4.6)			
**Chronic disease status (number of diseases)**									.29
	0	804 (40.0)		528 (41.3)		276 (37.8)			
	1	669 (33.0)		442 (32.8)		227 (33.4)			
	2	419 (18.6)		277 (17.6)		142 (20.2)			
	3	149 (5.5)		98 (5.8)		51 (4.8)			
	4	37 (1.7)		23 (0.9)		14 (3.0)			
	5	10 (1.3)		7 (1.6)		3 (0.8)			
**Search health information on the internet**									.002
	No	227 (12.3)		177 (15.1)		50 (7.4)			
	Yes	1845 (87.7)		1186 (84.9)		659 (92.6)			
**Insurance status**									.66
	Uninsured	63 (4.9)		41 (4.4)		22 (5.6)			
	Insured	2030 (95.1)		1338 (95.6)		692 (94.4)			

^a^N/A: not applicable.

Regarding the 3 engagement activities, disparities were also apparent between respondents who used the mobile app and those who did not. The mean VDT score for those who reported using an app was 1.56 (SD 0.83) compared to 1.11 (SD 0.71) for those who did not (*P*<.001). For PPI score, the average was 1.15 (SD 0.79) for those who used an app versus 0.87 (SD 0.81) for those who did not (*P*<.001), while, for the PPHI score, the average was 0.77 (SD 0.93) for respondents who used an app versus 0.48 (SD 0.77) for those who did not (*P*<.001). The significant *P* value for all engagement factors indicates that the use of engagement functionalities differed between users who reported accessing their portals through a health app and those who did not.

**Figure 1:**
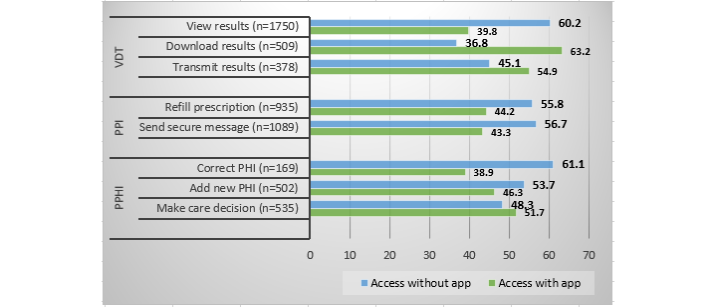
Comparison of usage by access type among users who claimed to have used personal health record functionalities. PPHI: patient–personal health information interaction; PPI: patient-provider interaction; VDT: view, download, and transmit.

Figure 1 shows that the usage of individual functionalities varied with access type. Among respondents who answered “Yes” when asked whether they used electronic medical records to make decisions about health care options, the number of participants who accessed their portal using a mobile app was higher (n=376, 51.7%) than that of non–app users. Similarly, the numbers of app users who downloaded medical results or shared records with a third party were also higher (n=196, 63.2% and n=288, 54.9%, respectively) than those of non–app users. Furthermore, web access was higher for other functionalities, including adding additional information to one’s medical records (n=278, 53.7%), correcting medical records (n=94, 61.1%), sending secure messages to providers (n=645, 56.7%), requesting prescription refills (n=559, 55.8%), and viewing medical results (n=1108, 60.2%).

**Figure 2:**
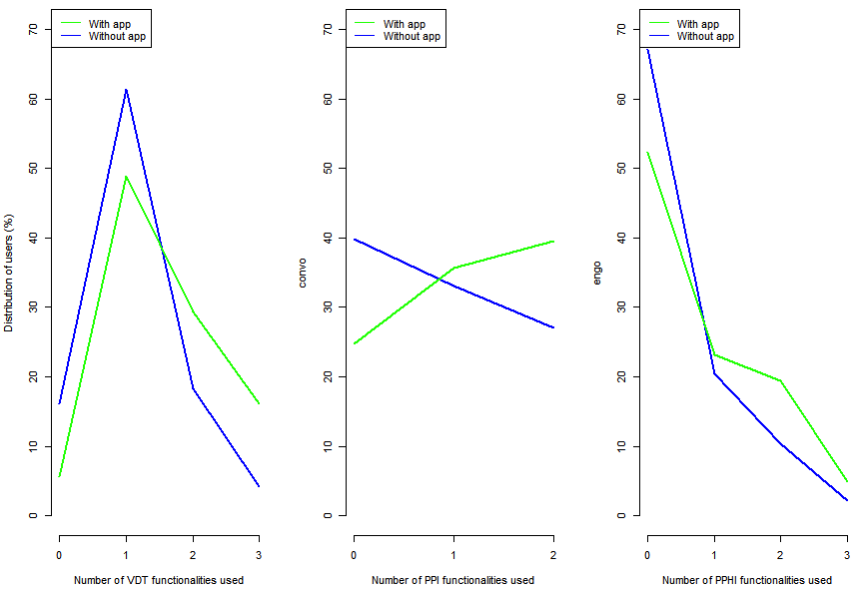
Comparison of the number of functionalities used by access type. PPHI: patient–personal health information interaction; PPI: patient-provider interaction; VDT: view, download, and transmit.

Figure 2 depicts the distribution of how the respondents used multiple functionalities. The usage of portal functionalities in general and the interaction functionalities, in particular, remained low. Nearly 54% (n=374) of app users and 70% (n=925) of nonusers did not engage in any PPHI task. Similarly, over 25% (n=177) of app users and 40% (n=549) of nonusers did not engage in any PHI task. The fraction of respondents who did not use any VDT tasks remained under 20% (n=263) among both app users and nonusers. Overall, app users were more likely to engage in at least one task within each outcome score.

### Generalized Linear Model

Results from the survey-weighted linear model are presented in Table 3. Models 1, 3, and 5 examine the effect of app usage without an interaction term. The results show that accessing portals using a health app is positively associated with higher usage of VDT, PPI, and PPHI functionalities. Specifically, app usage is associated with an increase of 0.52 (*P*<.001) in the VDT score, 0.24 (*P*=.006) in the PPI score, and 0.21 (*P*=.009) in the PPHI interaction score. These results indicate that for every 100 patients who use a health app to access their medical records, 52 more VDT tasks, 24 more PPI tasks, and 21 more PPHI tasks are performed. Models 2, 4, and 6 account for the interaction effects between age group and app use. The coefficients of the main independent variable (app use) changed slightly but not significantly (0.52 to 0.58 for the VDT score, 0.24 to 0.23 for the PPI score, and 0.21 to 0.23 for the PPHI score). The *R*^2^ value increased for the VDT score from 0.19 to 0.20 but did not change for the PPI and PPHI scores, indicating that only the VDT model is better explained when the model includes an interaction term.

Respondents who reported general health statuses of *good* (β=–.53, *P*=.03) and *excellent* (β=–.58*, P*=.04) were less likely to engage in PPHI tasks than those who reported poor health. Chronic disease status was associated with the usage of PPI and PPHI functionalities. The propensity to search health information on the internet was also associated with higher usage of VDT and PPI tasks. Insurance status, income, education, race and ethnicity, and gender were not significant.

**Table 3:** Regression results.

		Engagement functionalities (participants, n=1753)							
		View, download, and transmit score (95% CI)			Patient-provider information score (95% CI)			Patient–personal health information interaction score (95% CI)	
		Model 1^a^	Model 2^b^	Model 3^c^		Model 4^c^	Model 5^d^		Model 6^d^
Access type (reference: access without an app)		0.52^e^ (0.35 to 0.69)	0.58^e^ (0.39 to 0.76)	0.24^f^ (0.08 to 0.40)		0.23^g^ (0.04 to 0.42)	0.21^f^ (0.06 to 0.37)		0.23^f^ (0.07 to 0.40)
Age group of ≥65 years (reference: <65 years old)		–0.01 (–0.12 to 0.11)	0.11 (–0.02 to 0.24)	–0.01 (–0.14 to 0.13)		–0.02 (–0.16 to 0.12)	0.07 (–0.08 to 0.21)		0.11 (–0.07 to 0.28)
Male gender (reference: female gender)		0.11 (–0.02 to 0.24)	0.11 (–0.01 to 0.24)	0.07 (–0.09 to 0.24)		0.07 (–0.09 to 0.24)	–0.08 (–0.21 to 0.04)		–0.08 (–0.21 to 0.04)
**Race and ethnicity (reference: White)**									
	Hispanic	–0.07 (–0.33 to 0.19)	–0.07 (–0.33 to 0.18)	–0.02 (–0.22 to 0.17)		–0.02 (–0.22 to 0.17)	0.02 (–0.16 to 0.20)		0.02 (–0.16 to 0.20)
	Non-Hispanic Asian	0.37 (–0.07 to 0.81)	0.39 (–0.05 to 0.82)	0.12 (–0.33 to 0.56)		0.12 (–0.33 to 0.56)	0.61 (–0.09 to 1.30)		0.61 (–0.08 to 1.31)
	Non-Hispanic other	0.54 (–0.09 to 1.17)	0.54 (–0.09 to 1.17)	0.27 (–0.16 to 0.70)		0.27 (–0.16 to 0.71)	0.86 (–0.58 to 2.29)		0.86 (–0.59 to 2.30)
	Non-Hispanic Black or African American	–0.15 (–0.35 to 0.06)	–0.14 (–0.34 to 0.06)	0.05 (–0.19 to 0.29)		0.05 (–0.19 to 0.29)	0.20 (–0.01 to 0.42)		0.21 (–0.01 to 0.43)
**Education level (reference: less than high school)**									
	High school graduate	0.08 (–0.34 to 0.49)	0.07 (–0.34 to 0.47)	–0.35 (–1.00 to 0.29)		–0.35 (–1.00 to 0.30)	–0.22 (–0.68 to 0.24)		–0.22 (–0.68 to 0.23)
	Some college	–0.10 (–0.49 to 0.30)	–0.10 (–0.48 to 0.27)	–0.27 (–0.84 to 0.31)		–0.27 (–0.84 to 0.31)	–0.18 (–0.68 to 0.32)		–0.18 (–0.68 to 0.31)
	College graduate or more	–0.06 (–0.45 to 0.33)	–0.07 (–0.44 to 0.30)	–0.30 (–0.90 to 0.29)		–0.30 (–0.90 to 0.29)	–0.19 (–0.66 to 0.28)		–0.20 (–0.66 to 0.27)
**Income (US $; reference: less than US $20,000)**									
	20,000 to <35,000	–0.06 (–0.40 to 0.27)	–0.06 (–0.39 to 0.27)	–0.05 (–0.46 to 0.35)		–0.06 (–0.46 to 0.35)	–0.04 (–0.35 to 0.27)		–0.04 (–0.35 to 0.27)
	35,000 to <50,000	0.13 (–0.14 to 0.39)	0.12 (–0.15 to 0.38)	0.02 (–0.31 to 0.36)		0.02 (–0.31 to 0.36)	0.09 (–0.25 to 0.43)		0.08 (–0.26 to 0.43)
	50,000 to <75,000	0.08 (–0.19 to 0.35)	0.09 (–0.18 to 0.35)	0.19 (–0.15 to 0.53)		0.19 (–0.15 to 0.53)	0.09 (–0.23 to 0.42)		0.09 (–0.23 to 0.42)
	≥75,000	0.10 (–0.15 to 0.34)	0.10 (–0.14 to 0.34)	0.12 (–0.16 to 0.41)		0.12 (–0.16 to 0.41)	0.16 (–0.14 to 0.45)		0.16 (–0.14 to 0.46)
**General health status (reference: poor)**									
	Fair	–0.10 (–0.59 to 0.38)	–0.11 (–0.59 to 0.36)	–0.40 (–0.97 to 0.16)		–0.40 (–0.97 to 0.16)	–0.37 (–0.96 to 0.22)		–0.38 (–0.97 to 0.22)
	Good	–0.32 (–0.83 to 0.18)	–0.33 (–0.82 to 0.17)	–0.35 (–0.90 to 0.20)		–0.35 (–0.90 to 0.20)	–0.53^g^ (–1.00 to –0.05)		–0.53^g^ (–1.00 to –0.05)
	Very good	–0.18 (–0.68 to 0.33)	–0.18 (–0.68 to 0.32)	–0.27 (–0.82 to 0.29)		–0.27 (–0.82 to 0.29)	–0.44 (–0.94 to 0.05)		–0.44 (–0.94 to 0.06)
	Excellent	–0.29 (–0.79 to 0.22)	–0.29 (–0.79 to 0.20)	–0.43 (–0.98 to 0.12)		–0.43 (–0.98 to 0.12)	–0.57^g^ (–1.11 to –0.03)		–0.58^g^ (–1.12 to –0.03)
**Chronic disease status (reference: 0 diseases)**									
	1	–0.01 (–0.16 to 0.14)	–0.01 (–0.17 to 0.14)	0.24^f^ (0.07 to 0.41)		0.24^f^ (0.07 to 0.41)	–0.04 (–0.24 to 0.15)		–0.04 (–0.24 to 0.15)
	2	0.02 (–0.14 to 0.19)	0.03 (–0.13 to 0.19)	0.29^f^ (0.09 to 0.48)		0.29^f^ (0.09 to 0.48)	–0.03 (–0.22 to 0.16)		–0.03 (–0.21 to 0.16)
	3	–0.05 (–0.22 to 0.11)	–0.05 (–0.20 to 0.11)	0.20 (–0.08 to 0.48)		0.20 (–0.08 to 0.48)	0.08 (–0.21 to 0.37)		0.08 (–0.21 to 0.38)
	4	0.08 (–0.47 to 0.63)	0.06 (–0.48 to 0.60)	0.22 (–0.24 to 0.67)		0.22 (–0.24 to 0.68)	–0.08 (–0.52 to 0.36)		–0.09 (–0.53 to 0.35)
	5	–0.13 (–0.73 to 0.47)	–0.13 (–0.71 to 0.45)	0.46 (–1.20 to 2.12)		0.46 (–1.20 to 2.11)	1.17^g^ (0.27 to 2.08)		1.18^g^ (0.26 to 2.09)
Insurance status: insured (reference: uninsured)		0.28 (–0.13 to 0.68)	0.27 (–0.13 to 0.67)	0.13 (–0.46 to 0.73)		0.13 (–0.46 to 0.73)	–0.16 (–0.51 to 0.19)		–0.16 (–0.50 to 0.19)
Searched health information on the web (ref: did not search)		0.33^g^ (0.08 to 0.58)	0.33^g^ (0.08 to 0.58)	0.31^g^ (0.08 to 0.58)		0.31^g^ (0.07 to 0.55)	0.10 (–0.35 to 0.55)		0.10 (–0.35 to 0.55)
App use × age group		Reference	–0.43^f^ (–0.71 to –0.15)	Reference		0.05 (–0.29 to 0.39)	Reference		–0.15 (–0.38 to 0.08)
Constant		0.70 (–0.00 to 1.41)	0.69 (–0.01 to 1.39)	0.80 (–0.05 to 1.66)		0.81 (–0.05 to 1.66)	1.04^g^ (0.18 to 1.89)		1.03^g^ (0.18 to 1.89)

^a^*R*^2^=0.19.

^b^*R*^2^=0.20.

^c^*R*^2^=0.09.

^d^*R*^2^=0.19.

^e^*P*<.001.

^f^*P*<.01.

^g^*P*<.05.

### Interaction Effects of Age Group

**Figure 3:**
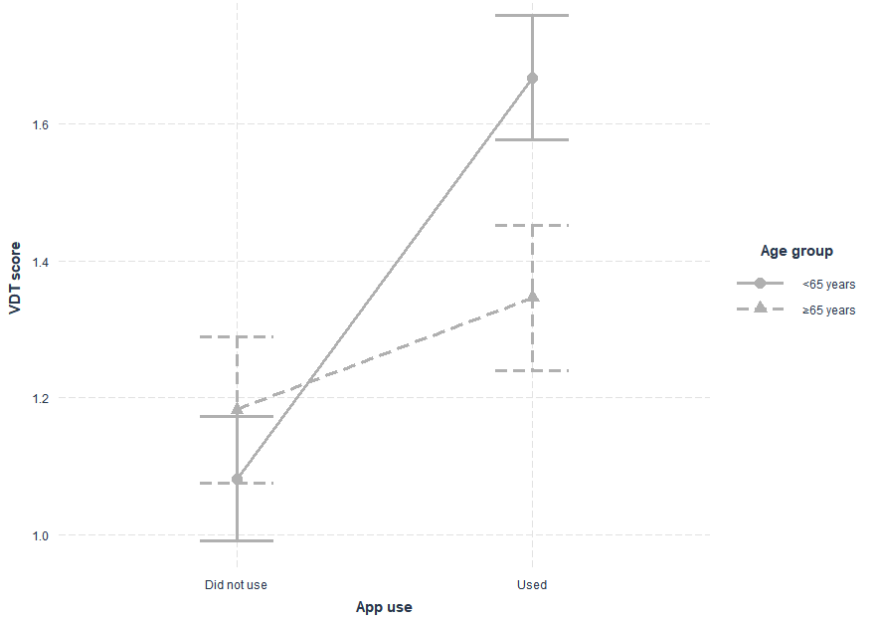
Interaction between functionalities and age. VDT: view, download, and transmit.

Models 2, 4, and 6 introduce an interaction term between app use and age group. The results indicate that the age group moderates the relationship (β=–.43, *P*=.004) between app use and VDT usage; that is, app use effects will differ between patients younger than 65 years and older patients. Table 4 and Figure 3 show the marginal means of VDT usage for all combinations of app use and age group. App users younger than 65 years exhibited higher averages than nonusers (1.67 vs 1.08, respectively). For users aged 65 years and older, Table 4 also shows a slight positive difference between app users (1.35) and nonusers (1.18). However, as shown in Figure 3, the mean difference is higher for users younger than 65 years, indicating an interaction between app use and age group variables.

The analysis of simple effects revealed a significant difference between those who used an app to perform VDT tasks and those who did not. The impact of app use was positive for both groups; however, the effect was significant only for participants younger than 65 years (β=.58, *P*<.001). Thus, app use was positively related to VDT use for users younger than 65 years but not for older users.

**Table 4:** Marginal means of VDT by app use and age group.

App usage	Age group (years)	Marginal mean (SE)
Did not use	<65	1.08 (0.0452)
Used	<65	1.67 (0.0639)
Did not use	≥65	1.18 (0.0528)
Used	≥65	1.35 (0.0952)

## Discussion

### Principal Findings

Health care delivery and outcomes can be improved with the increased participation of patients in health care decisions [44-47]. Portal functionalities, if sufficiently used, can be a driving force of patient-provider collaboration [4,14]. Thus, it is essential to integrate new and widely available technologies with portals to enhance patient engagement. To this end, our study has several significant findings. First, our study indicates that most portal users still do not use a health app. This finding aligns with the ONC report that states that only a small percentage of hospitals offers access to portals through health apps [11]. The positive effect of app use on patient engagement calls for more comprehensive portal mobile access functionalities. Second, regardless of how they access their medical records, many patients are still not engaging in any functionalities. Our findings show that the proportion of individuals who have been granted access privileges but do not access their portals is higher among non–app users.

Third, using a health app to access portals strongly and positively impacts the use of the multiple engagement functionalities. The regression coefficients of VDT, PPI, and PPHI engagement functionalities were positive and significant. Furthermore, Figure 2 shows that app users are more likely to use at least one engagement task and have higher usage of VDT, PPI, and PPHI tasks. This finding extends extant research that indicates that app users log into their portals sooner and more frequently than non–app users [41] by showing that beyond timeliness and frequency of use, the use of apps to access portals is associated with the breadth of the usage of portal functionalities. Fourth, the association of portal access type on individual portal tasks still favors non–app users. Portal usage of app users for individual tasks was greater only for 3 out of 8 functionalities, namely, using medical records to make care decisions, downloading, and transmitting results. Downloading and sharing medical records are associated with increased efficiency in clinical workflows [48]. Offering app access to patients who underuse these functionalities could help improve their portal usage.

Fifth, the impacts of mobile health app usage on the use of diverse functionalities fade with age only for VDT tasks. This finding is in line with that of previous research that the adoption of newer technologies and the breadth of use of technology tends to decline with age, even though users continue to frequently use technologies they are acquainted with [49]. However, this study shows that the age group of portal users does not condition the impact of access type on PPI and PPHI tasks. This finding extends previous research on the effects of age on mobile health app usage by showing that the result of mobile access is not uniform across all age groups and tasks. While mobile access increases VDT tasks for users younger than 65 years and decreases them, for others, it does not impact portal users differently on the breadth of use of interaction functionalities regardless of their age groups. App access increases the usage of interaction tasks similarly for all users. Mobile health app access could be a better intervention for portal users younger than 65 years on VDT tasks and all portal users for interaction tasks. Understanding the nuances in using portal capabilities is essential for providers to develop equitable interventions for each age group and to increase the use of underused functionalities. Other interventions, such as providers’ encouragement that have been associated with higher usage of portals [36], could be used among patients who are aged 65 years or older to increase the use of VDT tasks.

Logging into portals is only a first step toward patient engagement through their medical records. Once logged in, the number of tasks patients perform contributes to their empowerment and engagement. In this study, while a high percentage of users (46.5% and 62.8% of app users and non–app users, respectively) performed at least one VDT task, less than 20% of users in both groups engaged in all 3 VDT tasks. This low involvement with VDT tasks was more pronounced among non–app users. Similarly, less than 5% and less than 40% in each group performed all PPHI and PPI tasks, respectively. Additional research is needed to improve patient engagement with multiple portal functionalities.

Mobile apps provide convenience for their users [50]. For the most part, the effort required to perform VDT tasks is minimal. Therefore, VDT tasks can be more easily accomplished with a click or touch of a button for a health app designed with usability considerations. The same is not valid for interaction tasks that require the use of a computer or cellphone keyboard. Prior research comparing the use of smartphones with that of desktops in business settings shows that users of smartphones can perform reading tasks better than typing tasks [51]. However, the need to manipulate the keyboard and the smaller window size of a smartphone increase the time and difficulty of performing such tasks. Hence, performing interaction tasks via a health app is not as convenient as doing so via a computer and may explain the lesser impact of app usage on the interaction functionalities, as shown in Table 3. While app usage was significantly and positively associated with all engagement types, its impact on the VDT tasks was far more significant than that on the interaction tasks, as reflected in the regression coefficients. It is also possible that app interfaces were not easy to use to port data from one provider to another. Therefore, there is a need for apps to connect with platforms that make data portability seamless.

Even though app usage predicted all engagement types, the easier a task that could be performed via an app, the more it was used. Previous studies have recommended that portal designers simplify data entry into their systems [52,53]. App designers also should endeavor to decrease the amount of effort needed to perform interaction tasks, especially PPHI tasks, which are essential in this new era of interoperability across health information systems and information exchanges [19]. The ability of users to amend or request amendments to their records could be vital in reconciling fragmented information from one system to another and ensuring data integrity and completeness.

### Limitations

This study used secondary data. Thus, covariates were limited to variables available in the data. Similarly, this study was based on cross-sectional data and cannot infer causality. Future research could examine the impact of health app usage on the portal engagement functionalities using longitudinal data.

These secondary data did not provide details on the functional characteristics of the mobile apps and portals that were used. It was also unclear whether the portals were tethered to an electronic health record. Since the data were representative of the national US population [54], this study assumed that the mobile health apps and portals used by the study population were diverse.

### Conclusions

Patients with access to mobile-optimized portals log into their medical records sooner after being granted access and more frequently than those who only use a computer [38]. This study shows that patients who use a health app to access their records are also more likely to engage in multiple VDT, PPI, and PPHI tasks. The convenience and wide availability of health apps can also improve VDT functionalities among adults younger than 65 years and interaction functionalities among all portal users. Previous studies show that engagement with portals leads to better health outcomes and effective and efficient care delivery [21,22]. Although the likelihood of engaging in at least one task is higher when using an app, portal usage remains low. More research is needed to determine other factors and characteristics of health apps, which could lead to greater portal usage, especially among adults aged 65 years or older.

## Appendix

Multimedia Appendix 1 Survey questions and variable measurements.

## Abbreviations

EFA: exploratory factor analysis
HINTS: Health Information National Trends Survey
ONC: Office of the National Coordinator
PHI: personal health information
PPHI: patient–personal health information interaction
PPI: patient-provider interaction
VDT: view, download, and transmit

## Data Availability

All data analyzed during this study are publicly available on the HINTS website [54].
